# Full wave 3D inverse scattering transmission ultrasound tomography in the presence of high contrast

**DOI:** 10.1038/s41598-020-76754-3

**Published:** 2020-11-19

**Authors:** James Wiskin, Bilal Malik, David Borup, Nasser Pirshafiey, John Klock

**Affiliations:** QT ultrasound LLC, Novato, USA

**Keywords:** Medical imaging, Three-dimensional imaging, Tomography, Ultrasonography, Whole body imaging, Paediatric research, Translational research, Computational science, Health care, Medical research, Mathematics and computing

## Abstract

We present here a quantitative ultrasound tomographic method yielding a sub-mm resolution, quantitative 3D representation of tissue characteristics in the presence of high contrast media. This result is a generalization of previous work where high impedance contrast was not present and may provide a clinically and laboratory relevant, relatively inexpensive, high resolution imaging method for imaging in the presence of bone. This allows tumor, muscle, tendon, ligament or cartilage disease monitoring for therapy and general laboratory or clinical settings. The method has proven useful in breast imaging and is generalized here to high-resolution quantitative imaging in the presence of bone. The laboratory data are acquired in ~ 12 min and the reconstruction in ~ 24 min—approximately 200 times faster than previously reported simulations in the literature. Such fast reconstructions with real data require careful calibration, adequate data redundancy from a 2D array of 2048 elements and a paraxial approximation. The imaging results show that tissue surrounding the high impedance region is artifact free and has correct speed of sound at sub-mm resolution.

## Introduction

Ultrasound has been a useful tool in medicine since the post WW II era. However, there is presently no ultrasound tomographic system for orthopaedic or whole-body imaging in the presence of high impedance contrast (bone). Recent work with simulations has shown promise^1^. However, they bypass critical issues of calibration when dealing with in vivo data and the image reconstruction times given are not feasible clinically. They state they require a speed up of approximately 200 times.

Initial applications of ultrasound were relatively simple. However, beginning with early work by Greenleaf and Johnson at the Mayo Clinic in 1974–1980^2,3^, ultrasound tomography (UST) has developed into a useful modality for breast imaging. Original work by S. Johnson’s group at the University of Utah^4–8^ led to high contrast and spatial resolution images in 2008^6,8–12^, which were steadily improved, resulting in FDA clearance for its dedicated prone breast scanner in 2017^13^.

Another approach commonly employed in the literature is based on the ‘diffraction tomography’ approach of Devaney^14,15^. In this case a linear approximation to a nonlinear problem results in a canonical closed form solution to the ‘inverse scattering’ problem. The difficulty with this mathematically elegant approach is that the contrast and size for which it works are smaller than encountered in human tissue. Consequently, even in breast tissue imaging it has been felt to be inadequate, and in the presence of bone, the failing is more severe. We present here a fully nonlinear inversion of laboratory data. This nonlinear full wave imaging approach has been used in variant form in the time domain^16^, where a higher-order Born approximation is used to address the computational complexity of full inversion. This mathematically elegant extension beyond Born is carried out in 2D in the context of radar tomography. Also, in the context of ultrasound a projection based method using Krylov subspace expansion gives better results than the much slower singular value decomposition^17^. Another electromagnetic (EM) problem is the more difficult case where phase information is not available, where a contractual integral equation (CIE) is used with Contrast Source Inversion regularization^18^.

Our success with breast imaging has led to the desire to extend these results using the paraxial approximation with alternate frequencies and iteration counts to orthopaedic and whole or meso-body imaging in the presence of high impedance contrast, which is presented here for the first time to our knowledge. Furthermore, there is clinical need for sound wave imaging as described here because of 3 issues: (1) currently MRI has challenges directly measuring tissue characteristics such as cartilage, tendons, periosteum and the interior of trabecular bone (see below), (2) MRI has practical requirements of a high magnetic field and specialized facilities and (3) required special training and in some cases (potentially harmful) contrast administration. Quantitative ultrasound (QTUS) tomography could potentially address some of these issues as well as provide heretofore unavailable data, such as quantitative and accurate tissue characteristics measured at mm scale, in research and clinical settings. Other research groups have been developing alternative methods^19–36^ for breast imaging. The advantage of breast tissue for ultrasound tomography is the lack of high contrast tissue, such as bone. This makes the application of advanced ultrasound tomographic algorithms feasible^37,38^. There has been good clinical success in breast imaging with high sensitivity and specificity when compared to hand held ultrasound and mammography^39^.

A relevant tissue characteristic to acoustic wave propagation is its intrinsic impedance, which is roughly the product of mass density x speed of sound in tissue ($z = \rho c$). Soft tissue (such as is found in the breast) generally has a speed of sound found empirically proportionate to density^40^, and the variation is on the order of 10%. The relatively low contrast found here differs sharply with the much higher impedance contrast of tissue to bone, which makes quantitative ultrasound tomography difficult in these scenarios.

We present here a data acquisition system and reconstruction algorithm which gives the required reconstructions from laboratory data (not simulations) establishing the feasibility of ultrasound tomography even in the presence of bone. We note the ~ 200 times speed up that Guasch et al.^1^ require with a reconstruction time of ~ 24 min for the algorithm presented here. The data were acquired in ~ 12–14 min (depending on the rate of rotation of the arrays). This yields an apparatus and method that yields clinically and laboratory useful information at a high resolution not presently available^41^.

Furthermore, this technology addresses the issue at a cost of ~ 1/10 of existing similar MR images, and is relevant to personalized medicine and accurate tumor monitoring before or after initiating cancer therapy. Also, a high resolution map of soft tissue is valuable for research applications as well as clinical monitoring of Duchenne Muscular Dystrophy and verification/monitoring of skeletal muscle regeneration through decellularized muscle scaffolds and similar methods^42–45^. This technology allows machine learning (ML)^46^ and radiomics to apply to ultrasound tomography images even in the presence of bone. Machine learning has historically been applied to other ultrasound modalities^47–50^ and other images, but the presence of bone has lead to artifacts that make ML problematic. Our lack of such artifacts makes application of ML more fruitful. . We report here a method that gives quantitatively accurate estimates of the speed of sound of tissue proximal to bone from laboratory data in ~ 24 min (reconstruction time) and discuss clinical applications. This method also addresses high costs of imaging and corresponding unavailability to low income areas. It yields biomarkers (speed of sound, attenuation and reflectivity) previously unavailable at such high resolution^41,51^.

## Results

The inversion algorithm is similar to the inverse scattering algorithm for the breast^13^, which is a first level generalization of the Radon transform that includes both nonlinearity and diffraction information^13^. That algorithm maintains the projection-like geometry of Radon and diffraction tomography but includes nonlinear and extended refraction and diffraction effects. This algorithm, first proved in the context of the breast^51–53^, is extended to include bone by the careful choice of alternative frequencies and iteration counts at each frequency. This extension of the algorithm to these more difficult cases involving bone, is analogous to the use of different ‘sequences’ in MR imaging. The fundamental algorithm is similar, involving the paraxial approximation, and a full 3D model, but the unique use of particular data is critical to success, just as different MR sequences yield different results. This is part of the contribution of this paper.

We have shown the stability of our algorithm with laboratory data (not simulation) by re-imaging the same individual 10 times and estimating the volume of fibroglandular tissue as a percentage of total breast volume. We observed an average of 9.4% with a standard deviation of 0.2%. Since the ratio is based upon quantitative speed of sound^52^ this indicates the stability of the transmission ultrasound reconstructions.

We note that the speed of sound interior to bone may be incorrect. However, an ‘effective’ speed of sound may result from the linear combination of the SOS of the fluid and the matrix structure in trabecular bone. Further research is needed to show whether our measured interior SOS may represent such an effective SOS, or in fact may represent a slow compressional Biot wave that propagates through the trabecular bone. Regardless, we observe the quantitative accuracy of the SOS of ligaments, tendons, cartilage, muscle, fat and skin in proximity to this bone. The absence of artifacts indicates the forward model is appropriate. There are undoubtedly some mode conversion events, but the images indicate they may not be strong enough to destroy the quantitative accuracy of the reconstruction of surrounding tissue. This is unexpected in the presence of bone and gives clinically important images.

**Figure 1:**
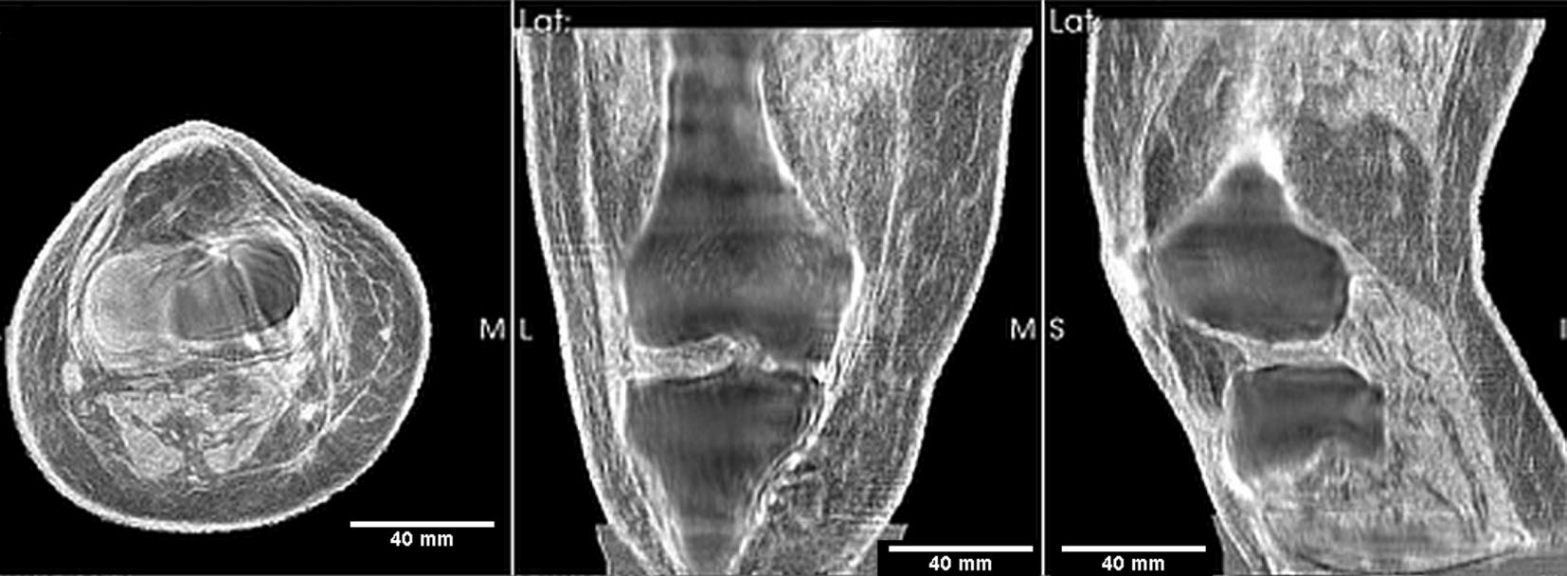
Fused image: axial, coronal and sagittal views of the transmission ultrasound 3D speed of sound map fused with reflection image of mature human knee. This representation highlights the 3D nature of the reconstruction. The articular cartilage in the tibiofemoral space is clearly visible. The lateral and medial collateral ligaments are visible as (correct) high speed regions. The high speed muscle is also shown with the correct SOS being read off of the perfectly registered pure SOS (speed of sound) image.

Figure 1 shows the 3D reconstructions of the fused speed of sound and reflection images of a human knee. The coronal image shows on the left the lateral collateral ligament and vastus with the correct (high) speed of sound. The high speed cortical bone/periosteum is also seen surrounding the apparent lower speed of sound (SOS) interior trabecular bone. We have pointed out the low speed of sound interior to the bone could be either an ‘effective’ SOS as an average of the SOS of the matrix and the fluid in trabecular bone, or it could represent the dominance of the slow Biot compressional wave. In either case, the accuracy of the soft tissue (cartilage, ligaments, tendon, muscle) surrounding the bone is not affected as documented below. This verification is the first step towards verification in the more difficult case when bone is present. We do note, however, that for orthopaedic/limb/extremity imaging we appear able to obtain a value close to the bone speed of sound.

### Separation of bone

A critical step in the imaging process is the clear separation of the bone (femur, tibia and fibula in this case) from the soft tissue, which is established via the attenuation image. We recognize that the SOS of our bone image is well below literature values. There are well known reasons for this including the high impedance contrast which greatly reduces the SNR of signal passing through the bone^54^. Furthermore, signal passing into the bone is subject to mode conversion that may not be correctly modeled by the scalar Helmholtz equation. The compressional wave incident upon the solid matrix of the bone may excite shear waves.

### Trabecular versus cortical bone

We note that there are two types of bone: trabecular or cancellous, which has the characteristic ‘spongy’ appearance. Hard bone forms a matrix in which there is fluid—marrow and blood. The cortical bone is thin at the epiphysis—i.e. at the extremities of the long bones that make up the human frame, and thus we are able to image in the tibiofemoral space, and believe energy is propagating through the bone at the low frequencies. See also^1^ for indications that energy is penetrating bone.

Also, there is independent evidence that the porous nature of the trabecular bone and thinness of the cortical bone near the tibiofemoral space will result in propagation of acoustic energy into the porous medium (trabecular or cancellous bone) and subsequent generation of a ‘slow’ compressional wave via Biot’s model^55^. This Biot model produces a ‘slow compressional wave’ having a speed of sound substantially less than the standard compressional wave^56–59^. There has been evidence that this slower wave has a larger amplitude than the standard compressional wave^60–62^. These studies were carried out on human femoral bone at frequencies relevant to our work (1 MHz). These observations may lead to clinically useful data related to osteoporosis.

The production of the quantitative speed of sound and attenuation image in the presence of the femur is a multiple step process:

1. Inverse scattering reconstruction of the speed of sound and attenuation images
2. Morphological post-processing on the attenuation image to isolate the bone
3. Fusion of the resulting attenuation with SOS and reflection images.

**Figure 2:**
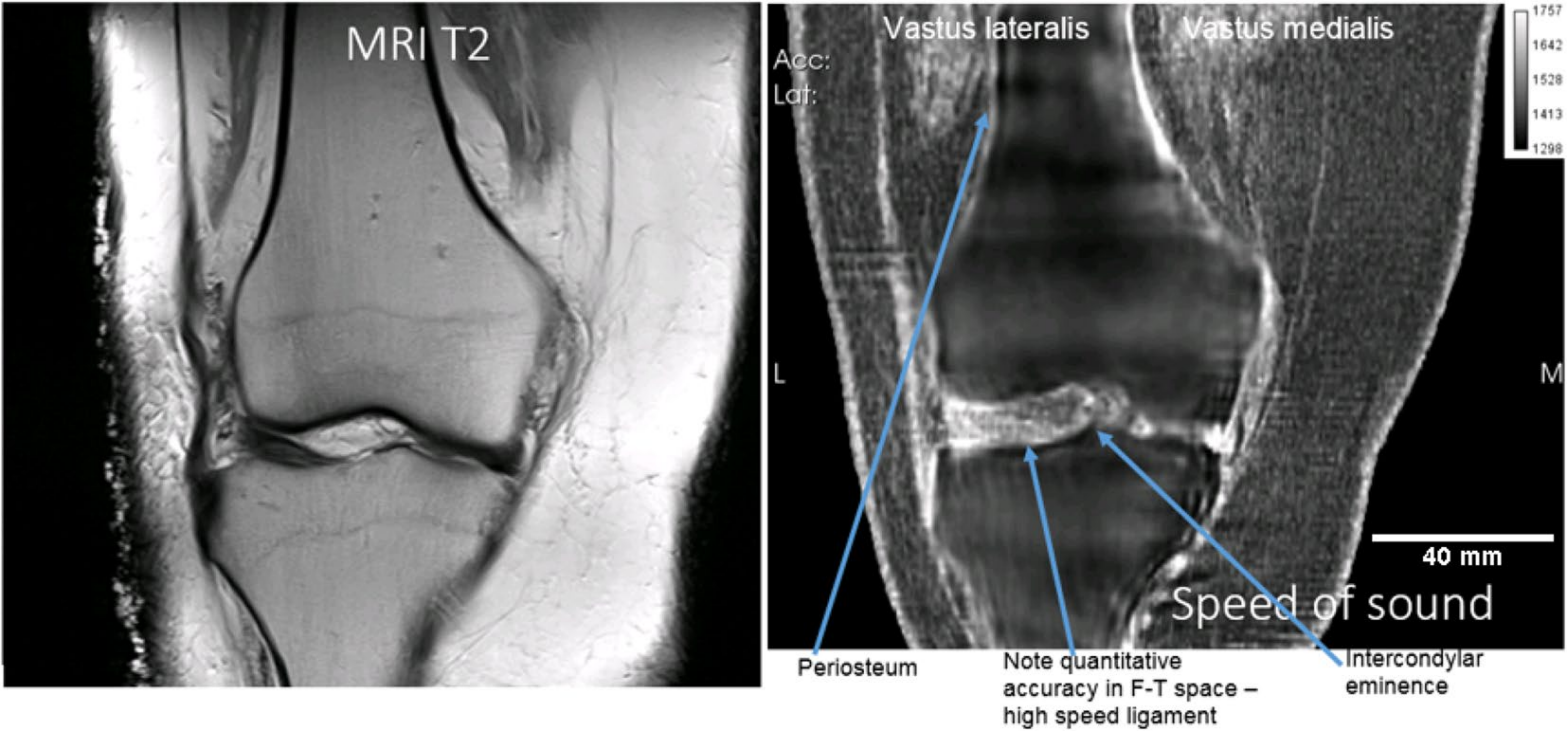
Showing correlation of speed of sound (right) and MR images (left) for the coronal view of the cadaver knee for T2 weighted MR image on the left versus SOS image on the right. Note the periosteum has the correct high speed and is clear on the QT ultrasound SOS image (right) whereas it does not generate a signal in the MR image (left panel). The lateral collateral ligament is likewise visible with correct SoS on the right but does not generate a signal in the MR image (left).

The fusion is a linear combination of speed of sound, attenuation and reflection, of the form $I_{ijk} \equiv \alpha (SoS)_{ijk} + \beta (Atten)_{ijk} + \gamma {\text{(Refl)}}_{ijk}$, for $\alpha ,\beta ,\gamma \in R$, real numbers, and *i,j,k* are voxel labels. Typical values are $\alpha = 1000,\beta = 100,\gamma = 6$, and also vary with units used. This is a large area of research and these values are not optimized. This does not negatively affect our ability to measure quantitative speed of sound at high resolution, since the images are correlated directly, which allows the direct reading of the correct speed of sound from a correlated pure SoS image. Figure 2 shows the articular cartilage and ligaments interior and close to the tibiofemoral space are imaged with correct speed of sound (high).

In particular the lateral collateral ligament has a high speed of sound in the right panel, left side of image. The intercondylar eminence is visible and the articular cartilage is clearly visible in the tibio-femoral space. This shows SOS is high for the articular cartilage, corresponding to literature values as shown in Table 1, and we measure the thickness of the articular cartilage at ~ 1.9 mm which agrees with literature.

**Table 1:** Comparison of speed of sound (SOS) for muscle fat and cartilage, with known literature values^54,63^.

Tissue	SOS QTUS 3D (SD) (m/s)	SOS (SD) (m/s)
Vastus medialis	1573 (25.9)	1588.4 (21.6)
Fresh muscle (chicken)	1572.6 (14.6)	1588.4 (21.6)
Fat	1438.7 (20.5)	1440 (21.4)
Cartilage	1655.4 (14.6)	1660

### Quantitative speed of sound measurement

This technique allows us to quantitatively image muscle, cartilage, tendons, ligaments, nerve bundle tissue, veins in the presence of bone. Acquiring quantitative accuracy is important and obtained by minimizing the ‘volume averaging effect’, wherein adjacent voxel values are smeared together yielding inaccurate values.

**Figure 3:**
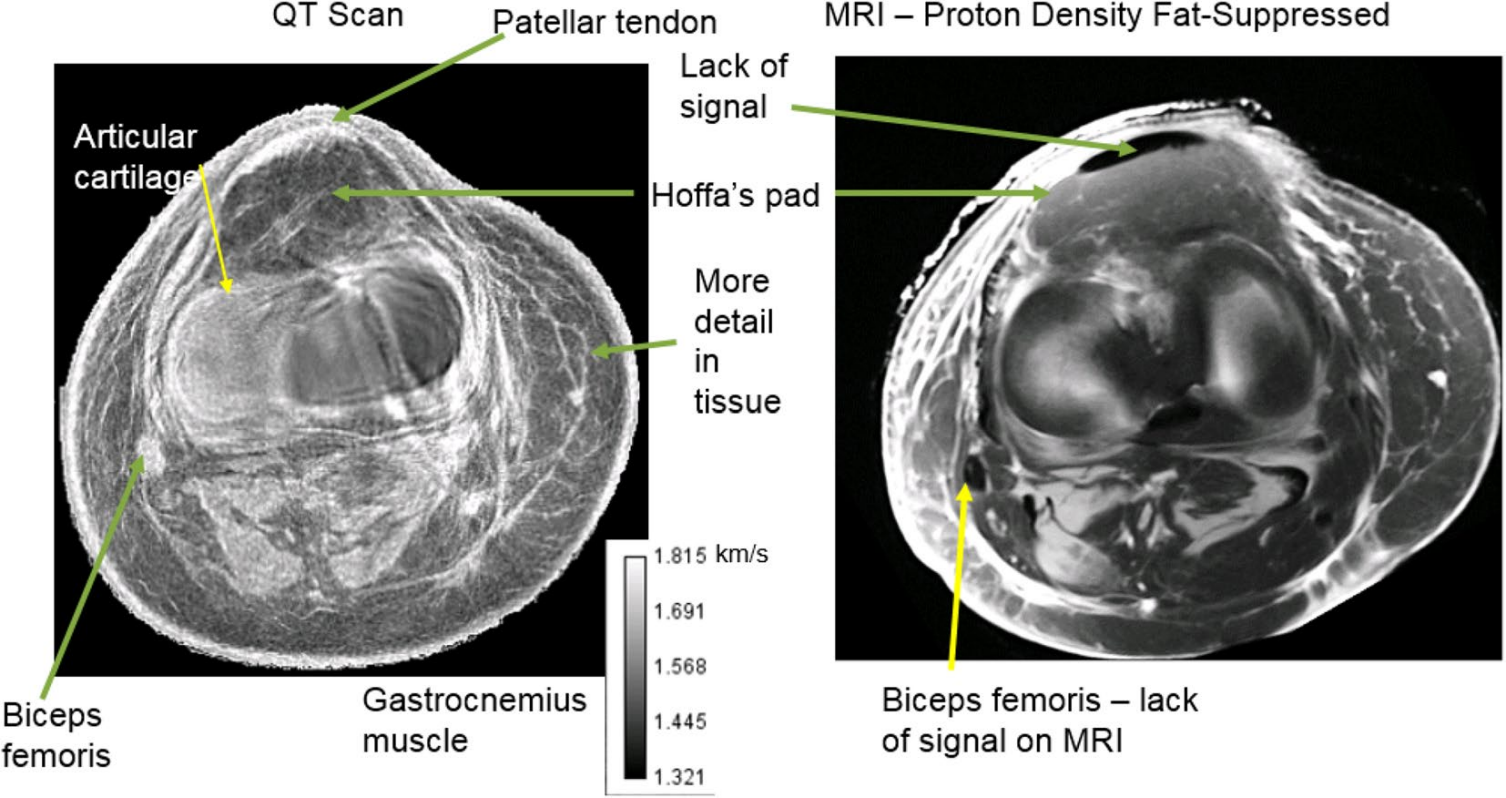
Comparison of the speed of sound image (left panel) with MR image of the same level. Note that the patellar tendon is invisible with this particular sequence for the MR image. The biceps femoris is also lacking signal from the MRI. Contrary to this, both the patellar tendon and biceps femoris have the correct quantitative speed of sound (high) for the SOS image. As noted in the text, there are structures in the QTUS image that are not visible in the MR image. We get quantitative values for the patellar tendon with QTUS, and no signal from the MR image, for example.

An important ability of our scanner is to monitor subtle and localized changes in the speed of sound of cartilage, ligaments, tendon or muscle. This is critical for myopathic monitoring of tissue health in general. As in Fig. 3 where the axial view comparison of the human knee cadaver: (left panel) shows the fused SOS and refraction corrected reflection image. The sub-mm resolution of the connective tissue is clearly shown, and the quantitative values of the fat (low SOS), patellar tendon, muscle/tendon complex (Biceps femoris) and muscle are clear on the left panel. On the other hand the proton density fat-suppressed image on the MR image—right panel–shows lack of signal for the patellar tendon. Similarly the biceps femoris muscle tendon complex does not produce a signal in the MR image. This lack of quantitative accuracy in the SOS prevents this modality from being used to monitor any disease of the tissue.

20 cross sections (axial view) of the leg were taken and 6 mm diameter ROI’s were used within to estimate the speed of sound for muscle (Vastus medialis), fat, and cartilage. These values were compared with the “IT’IS Database”^63^, along with the standard deviations (among the 20 ROI’s) for each category. The measured thickness of the articular cartilage was ~ 1.9 mm and agrees with literature values.

Independently, we measured the average SOS within a vertical (coronal view) ROI for each of the following tissue types:

1. Articular cartilage
2. Medial collateral ligament
3. Sartorius muscle
4. Fat

The ROI varied in diameter to fit well within the particular tissue type being measured. Here the values were compared with those in Duck et al.^54^ and listed in Table 2.

**Table 2:** SOS values as measured in our viewer for 6 mm diameter ROI’s located at cross-Sects. 2 mm apart.

Tissue type	Measured SOS (m/s) (std dev)	Literature values (m/s)
Articular cartilage	1664 (26.5 m/s)	1660 (m/s)
Medial collateral ligament	1690 (38)	1725–1750
Muscle (sartorius)	1571 (28 m/s)	1560–1625
Fat	1437 (18.5)	1350–1480

**Figure 4:**
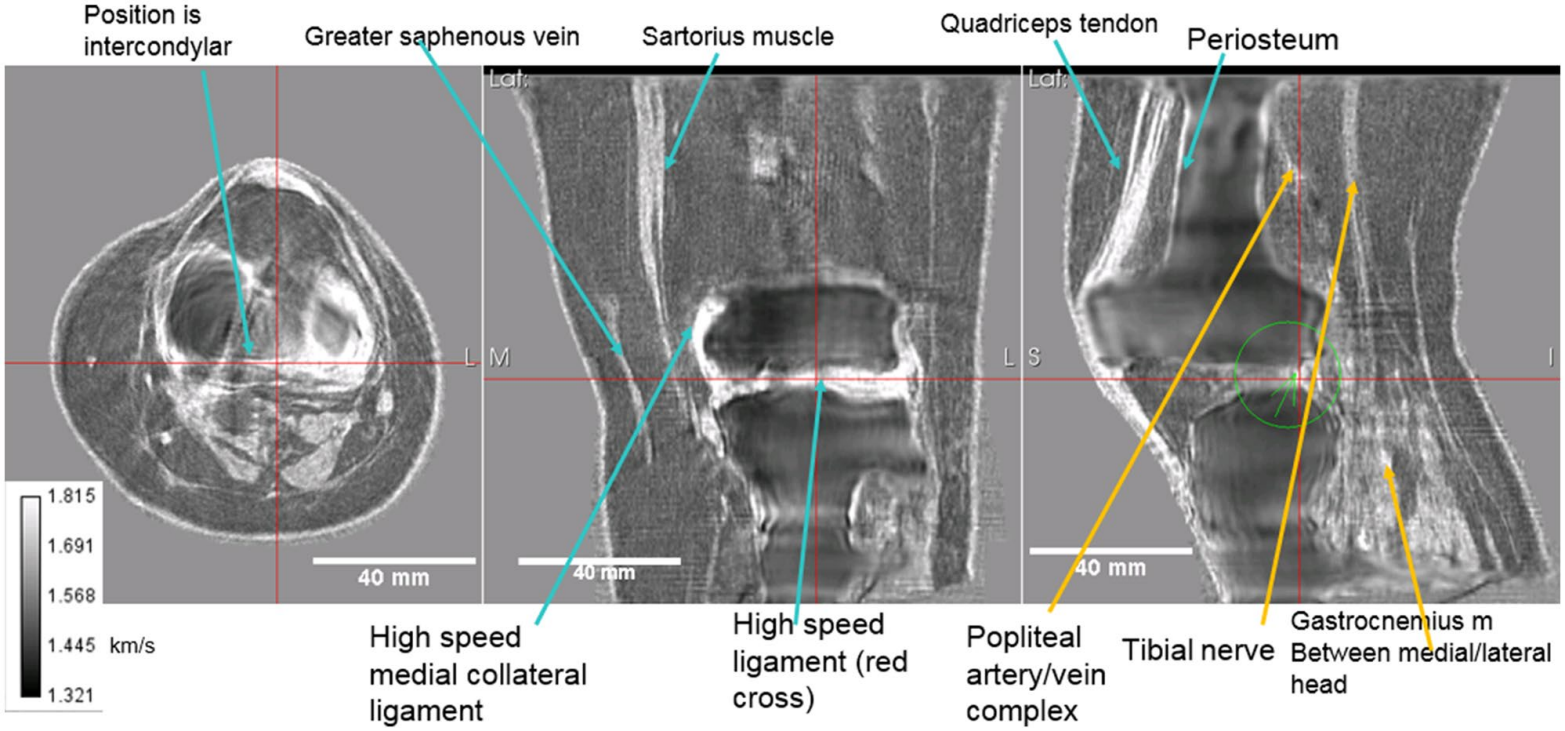
Speed of sound reconstruction showing full 3D nature of reconstruction. Left to right panels are: axial, coronal, sagittal. Note the reduced artifacts and correct speed of sound of the medial collateral and lateral collateral ligaments, the muscles, nerves (tibial nerve labelled) and artery vein complexes (saphenous vein and popliteal artery/vein complex shown). The fat and skin also have the correct speed of sound values. See text for further discussion.

Figure 4: shows the speed of sound image alone. The axial view is at the tibiofemoral level as can be seen by comparing the correlated images at the red lines. The gastrocnemius muscle (lateral and medial head) is seen in this view. The high speed patellar tendon also shows clearly. The coronal view shows the great saphenous vein with appropriate high speed of sound (although not as high as the medial collateral ligament, which is also seen as very light color). Note also the sartorius muscle in this view, and the tibiofemoral space itself with high speed articular cartilage. The quadriceps tendon is shown in the sagittal (rightmost panel), as well as the popliteal artery/vein complex and tibial nerve. The periosteum shows up as a high speed region as well.

**Figure 5:**
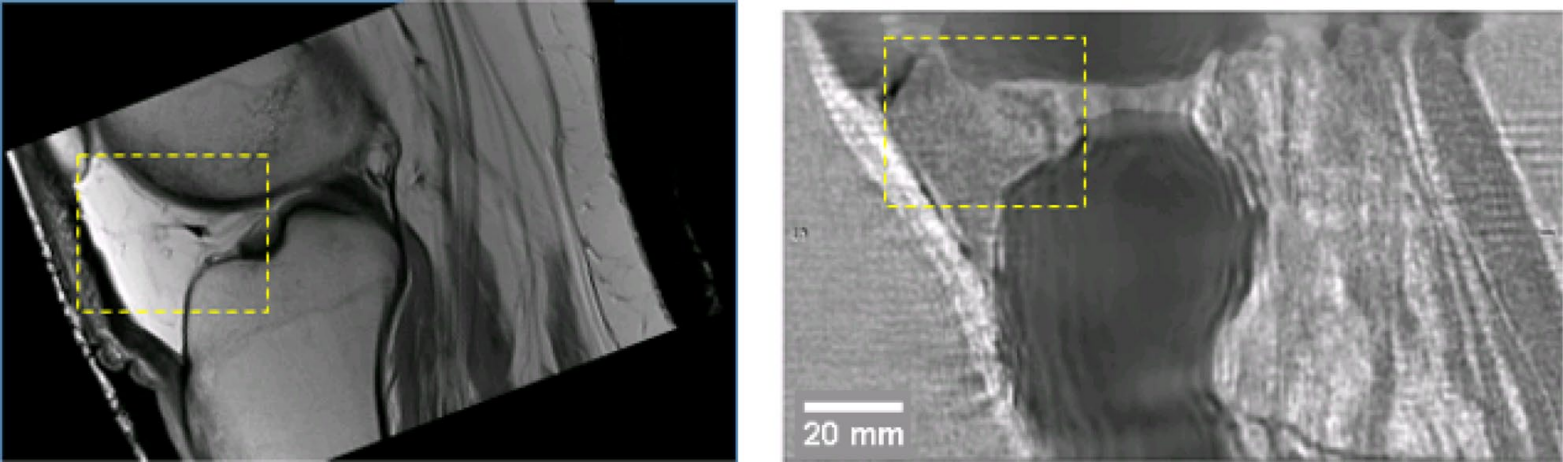
Comparison of the MR image (left) with the speed of sound image (right). The area enclosed in the yellow rectangle shows the Hoffa’s pad in both modalities. The correspondence is clear, even to the interface between the fat pad and the fat behind it, in the tibiofemoral space. The bone is clearly visible in the QT ultrasound image (QTUS). The high speed (light gray) muscle is seen in the SOS image and corresponds to the muscle showing up as a dark region on the MRI. The fat is light on the MRI, and dark (slow speed) on the QTUS image. The values from the QTUS image are quantitative correct to sub-mm resolution as shown in Tables 1 and 2.

Figure 5 shows the remarkable agreement between the MR image (left) and the QT ultrasound (QTUS) image (right). Note especially the region within the yellow dashed square as it shows an interface visible in both the MR and QTUS images. There is also correspondence in the bone structure, and the high speed tendons. However the tissue characteristics of the tendon are not evident on the MR, as they are in the QTUS image.

**Figure 6:**
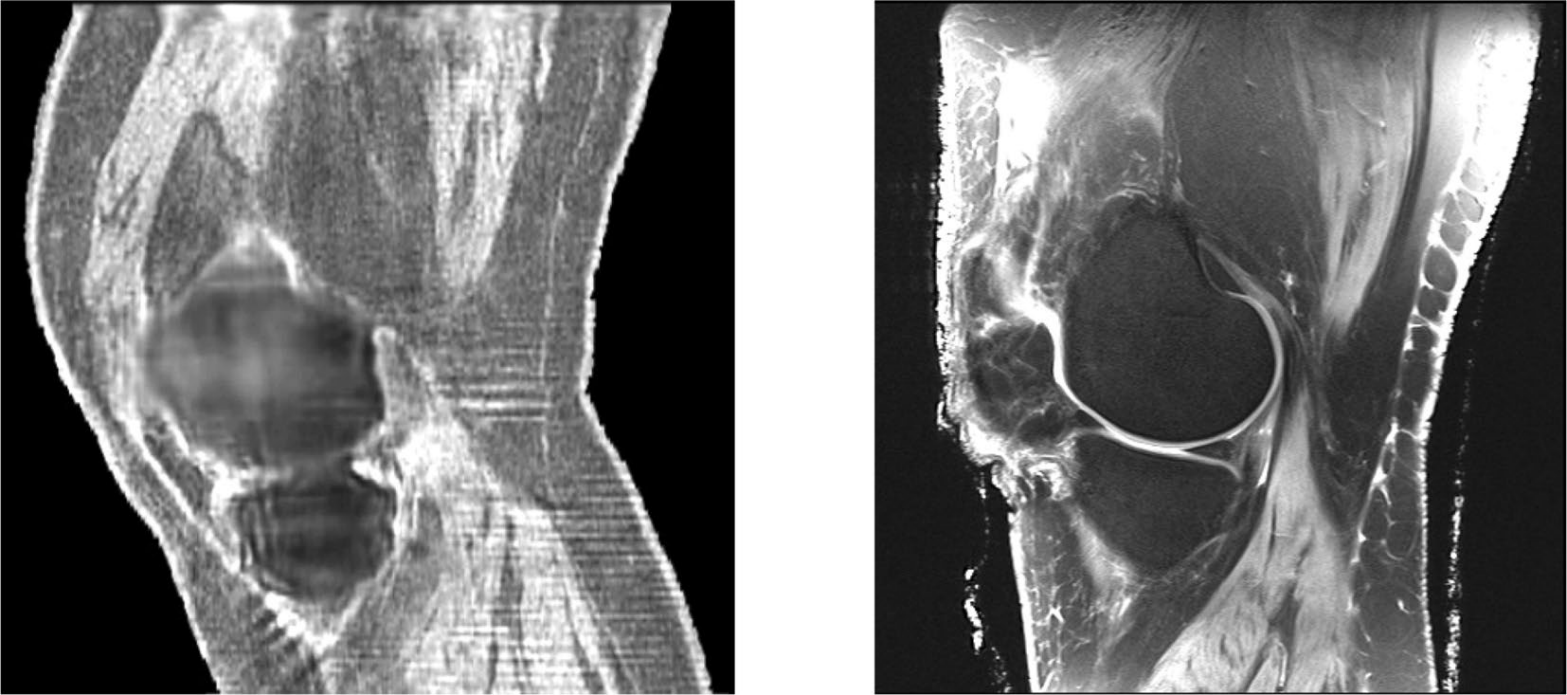
Sagittal view of knee: QTUS image on the left, MR image on the right. Note the close correspondence in the muscle tissue, bone, cartilage and patellar tendon and Hoffa’s fat pad. The bone is delineated.

**Figure 7:**
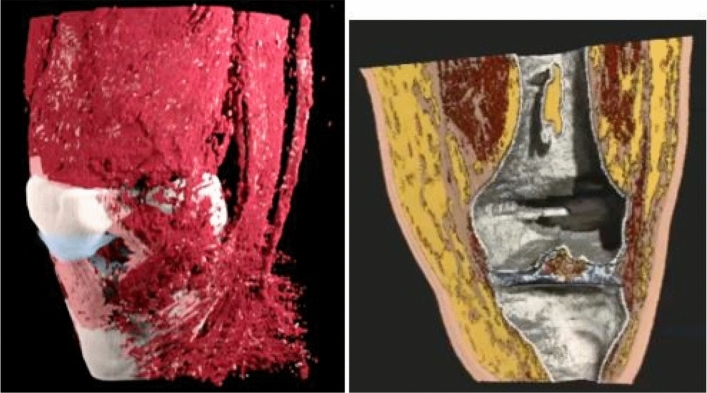
Left panel: 3D rendering of the SOS reconstruction of the human knee. See supplemental movie file_1 for motion. Here and in movie the fat has been suppressed. This shows clearly, the advantage of the quantitative values that allow direct segmentation of the different tissue types.The articular cartilage is blue, the muscle is red, the sub-patellar tendon and other ligaments are pink. The bone is ivory. . Right panel: Alternative colorization of segmentation based on the speed of sound image provided by Lightstream Technologies (courtesy J. Helms). The fat is shown as yellow, the skin as pink, the muscle tissue is red, and the articular cartilage is blue. Ligament structures, such as the medial collateral ligament visible on the left is brown. The bone is gray and hollow. There is clear separation in the tibio-femoral space. See Supplemental Movie 2.

Figure 6 shows a corresponding sagittal section of the knee. The muscle groups (gastrocnemius below and vastus above) are visible and correlate. Note also the correct (high) value for the speed of sound of the skin in the QTUS image. Fig. 7 shows the 3D segmentation that results from the quantitative values for the speed of sound. On the left fat is transparent, tendons and ligaments are pink and muscle is red. On the right fat is yellow, ligaments are brown, muscle is deep red, skin is pink and bone is white–gray on both. These are shown in 3D in the supplementary videos 1 and 2 respectively, which give the complete 3D nature of the segmentation and quantitative reconstruction.

**Figure 8:**
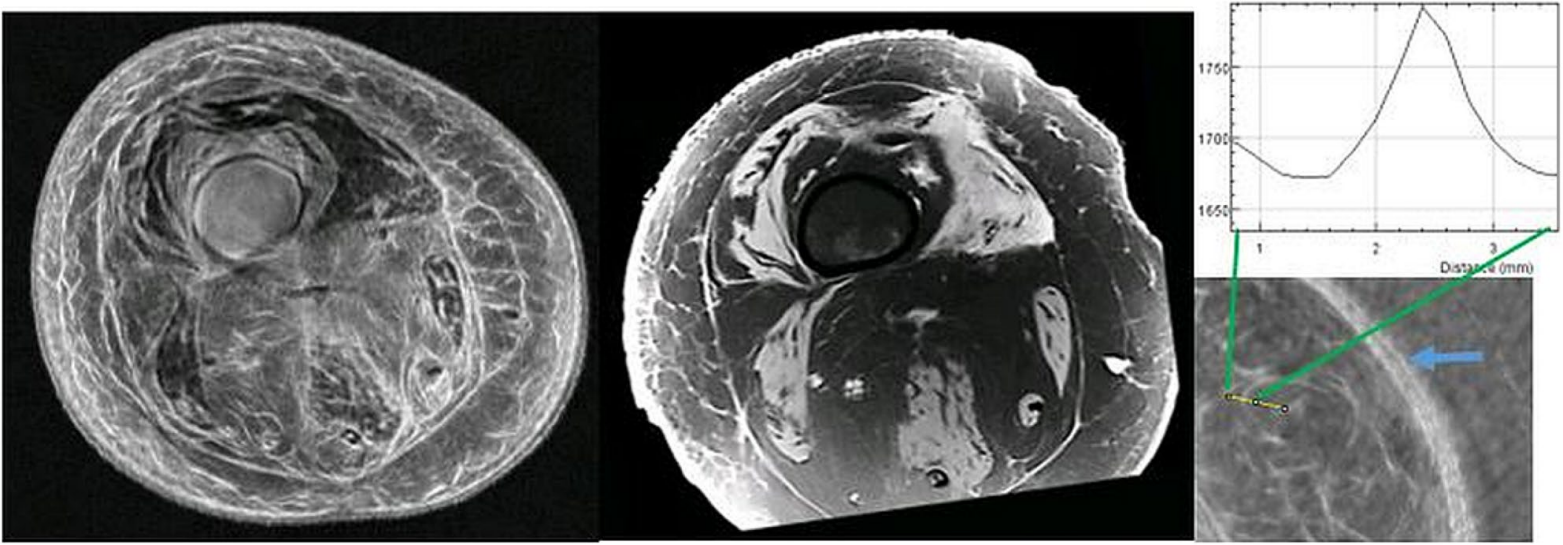
Left panel is speed of sound (inverted grayscale) fused with reflection image (axial). Middle panel is MR image (axial). A direct correspondence is seen between the vastus lateralis and vastus medialis muscle groups between the two images. The Sartorius, semimembranosus, biceps femoris muscles are seen to correspond in both images. The gray-scale is inverted for the SOS fused image on the left so the muscle groups appear dark in this image. The QTUS shows more detail than other color schemes due to highlighting of connective tissue. A close up of a fused QTUS knee image (axial) on the right shows also a cross-section (yellow bar), showing the thickness of connective tissue is 0.6 mm full-width half max (FWHM). Notice also the complete separation between dermis, epidermis, hypodermis in the right panel closeup. (blue arrow).

Further agreement between QTUS and MRI is seen in Fig. 8 showing axial QTUS (left panel) and MRI (middle panel) reconstructions of the cadaveric knee. Note the grayscale is inverted on the fused QTUS image (left panel) to highlight the connective tissue. Thus the muscle tissue is dark on the QTUS image whereas it is light gray for the MR image. Note also the close-up view of the QTUS fused image (rightmost panel) shows a line plot (yellow-bar). The line plot of the speed shows a full width half-max (FWHM) resolution of 0.6 mm. The pixel size is 0.2 mm. Note also the close-up view shows clearly the dermis, epidermis, hypodermis layered structure.

### Formalin fixation slightly affects SOS values

The images are derived from a cadaver knee, so we verified that the effect of formalin fixation had a minimal effect on tissue speed of sound. This is required to show clinical relevance of the device. To test this hypothesis we imaged fresh cadaver (unfixed) tissue, fixed the cadaver knee in formalin, then imaged the knee again. Note that speed of sound, $c = \sqrt {{K \mathord{\left/ {\vphantom {K \rho }} \right. \kern-\nulldelimiterspace} \rho }}$, where *K,* is the compressional bulk modulus, and *ρ* is the mass density. There is slight shrinkage of the tissue with the formalin fixing process, leading to increased mass density. However, there is also an increase in stiffness (*K*) *due* to cross linkage. The increase in both quantities means that the projected increase in *c is* expected to be small due to the $c = \sqrt {{K \mathord{\left/ {\vphantom {K \rho }} \right. \kern-\nulldelimiterspace} \rho }}$ formula. Thus we expect the reconstructed SoS values to be close to in vivo values, and this is what we observe as shown in Table 3, which shows the % change in fixed versus non-fixed cadaver knee tissue types.

**Table 3:** Summary of changes in different types of tissue observed in human breast after fixation with formalin. The effect is small due to changes in both mass density and compressional bulk modulus. See text.

Tissue type	Fixed versus non-fixed change (%)
Fat	+ 0.4
Skin	− 0.3
Fibroglandular tissue	+ 0.1

### Reconstruction times

A single iteration of the algorithm in the Ribiere–Polak nonlinear conjugate gradient technique is equivalent to 5 forward problems: Two forward problems for the analytic Jacobian calculation (also known as Frechet derivative) and two equivalent forward problems (‘adjoint field and backpropagated field) for the gradient calculation and a forward problem to check the residual with the new value. The efficiency of the forward problem and gradient formation lead to image creation in ~ 20 min, which is the speed up asked for in Guasch et al.^1^. Further this is done with laboratory data, which addresses the calibration problem which is handled as in Wiskin et al.^13^. This is a minimization of approximately 35 million unknowns, using ~ 22 GB of transmission and ~ 9 GB of reflection data. The actual times vary with the size of the tissue section being scanned. The quantitative accuracy of the image is maintained and there is a relative lack of degradation even with missing or degraded data, which is due to the massive redundancy of our data. This requires more research to quantify.

### Bone imaging protocol

**Figure 9:**
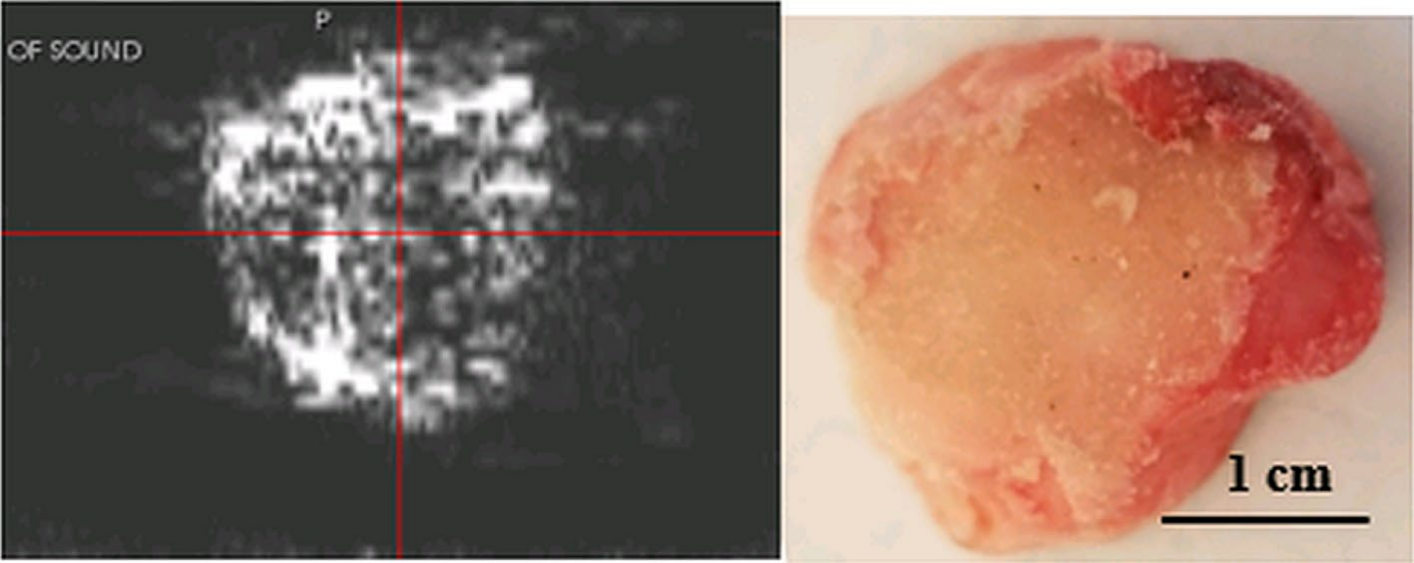
left panel ultrasound tomography (attenuation) image of cross-section of bovine bone. Right panel shows the optical image of the cross-section sawed froma bovine femur. This shows evidence that the trabecular part of the bone is visible in attenuation images due to the prevalence of edges. Note the attenuation is almost zero in the cortical part of the bone. The energy loss at the interface is interpreted as attenuation making the edge visible.

We note that the attenuation image of separate bone segment shows the trabecular nature of the bovine femur bone sample as seen in Fig. 9. This does not show up in the human case in the present cadaver, due to the surrounding tissue in the knee which attenuates the signal. However, this experiment shows that it may be possible to image the interfaces with lower frequencies and/or higher energies in the human knee.

**Figure 10:**
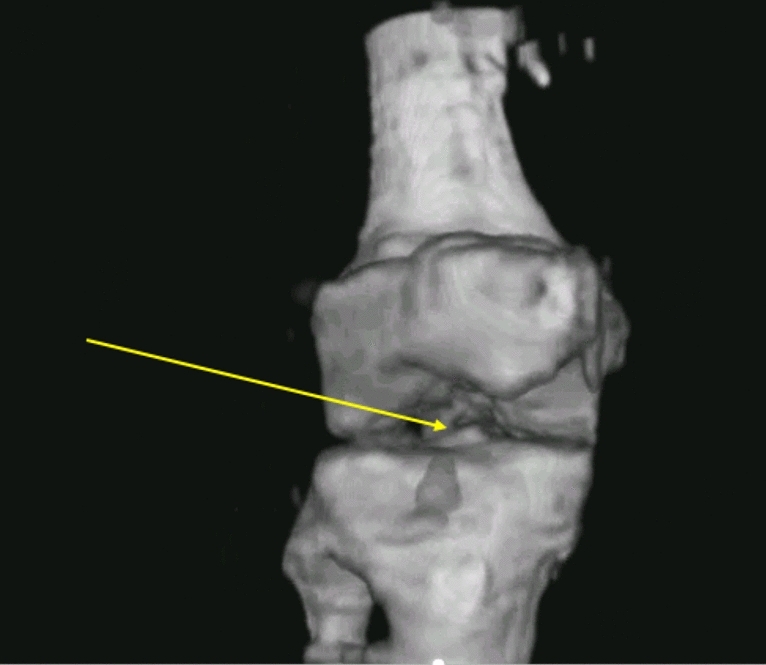
Coronal view of the tibiofemoral space showing the fibula, tibia, femur, tibial plateau with intercondylar eminence (yellow arrow) and the condyles themselves. See text for explanation and discussion. See supplemental movie 3 for the 3D version of this.

While we are not presently able to see inside the bone in vivo, the correct delineation of the bone is one of the more remarkable features of the particular sequence (frequencies used and concomitant iteration count) we used for orthopaedic imaging. This is seen in Fig. 10 which displays clear separation in the tibiofemoral space. In fact, the coronal view of the tibiofemoral reconstruction in Fig. 10 shows the clear separation and the intercondylar eminence on the tibial plateau. The condyles themselves are clearly visible as well. This image has been post-processed with morphological algorithms (opening, i.e. erosion followed by dilation). See the movie in ‘supplemental material’ for additional insight.

## Discussion and conclusion

Transmission ultrasound imaging has high spatial/contrast resolution and is safe for and broadly applicable to human and whole body medical and research imaging in the presence of bone. It has the following advantages: (1) it is inherently safe using low frequency and low energy sound, (2) it is fast and efficient, (3) it is less expensive than comparable imaging technology, making it available for low-resource areas—while providing high-quality medical/clinical and laboratory images (4) it meets all the needs of a point-of-care device for trauma and sport injury, (5) it can be adapted to partial-angle imaging—creating an “open” scanner design for intervention or biopsy, (6) it does not require a contrast agent or ionizing radiation, (7) it allows radiomics and machine learning analysis to be applied to the quantitative ultrasound tomography image, (8) it is aligned with personal precision medicine paradigms—allowing immediate monitoring of adjuvant/neo-adjuvant therapy. The quantitative values measured in the SOS image have been validated against literature values. Even the interior of the tibiofemoral (TF) space is visible with these frequencies. Within the TF space the articular cartilage is visible and quantified (correct SOS and thickness).

Refraction corrected reflection imaging is effective in showing high resolution speckle free representation of connective tissue at sub-millimeter resolution. The fusion of this mode with the registered SOS image yields a high-resolution map with complementary diagnostic information.

The images are reconstructed in approximately 20–40 min for ~ 20 cm of leg due to the incorporation of the paraxial approximation in the forward problem, the Jacobian calculation, and the associated adjoint required in the gradient calculations, and correct use of frequencies and iteration counts. Please see the Methods section for more detail regarding frequencies. This is for the full volume 214 mm by 214 mm by 192 mm tall. The data acquisition is in ~ 14 min.

The following specific examples of applications of quantitative ultrasound (QTUS) imaging are not exhaustive.

1. Quantitative tumor monitoring: (QTM) Monitoring the efficacy of cancer treatments relatively quickly due to the high accuracy and stability of the measurement of the lesions we observe. Changes in tumor size are measured volumetrically and are perfectly registered by virtue of the quantitative accuracy, repeatability and high resolution of the SOS and reflection images. A volume shrinkage of even a few mm’s will be quantifiably logged by our scanner. The safety of the scanning procedure means that a tumor can be scanned every week if desired. (There is no contrast agent, or ionizing radiation).
2. Myopathy monitoring: Duchenne muscular dystrophy (DMD) and the allelic phenotype Becker muscular dystrophy (BMD) are diseases that can be monitored non-invasively by QT images. Any treatment can be monitored for efficacy directly.
3. Preventative proactive tissue monitoring: The ability to quantitatively estimate in high resolution the speed of sound of muscle, cartilage, tendons, ligaments even in the presence of bone results in the capability to monitor strained ligaments/tendons that may be susceptible to breakage in horses, or humans in athletic endeavours, thereby preventing more serious damage or breakage. Such breakage is a serious problem in the thorough-bred horse-racing industry, where several thousand horses are put down every year due to such injuries, for example. It also enables correct monitoring of tissue changes when subject to proposed treatments.

While we do not claim to accurately measure the SOS of bone presently, future research is required to determine if we are indeed measuring the compressional slow Biot wave predicted by various modern reformulations of the original Biot theory. Furthermore, we have preliminary evidence that we are able to measure an ‘effective’ speed that may measure a linear interpolation between the speed of the bone matrix and the fluid in trabecular bone. The important point is the lack of artifacts introduced into the tissue part of the reconstructions. Note the imaging of meso-body images (large knees, neonates) allows the imaging of extremities, newborns and limbs without high magnetic fields or ionizing radiation.

We believe we have shown that the combination of full wave speed of sound transmission tomography and refraction corrected reflection imaging is poised to become a true whole-body imaging modality in the laboratory and clinic. This is especially true with the implementation of the paraxial approximation and the concomitant speed up and the highly redundant fully 3D data acquisition system. Finally we observe that the speed up of ~ 200 times that Guasch^1^ predict and desire for their algorithm would yield a reconstruction time of 6–12 s for this algorithm, further increasing the utility of this imaging modality for laboratory and clinical research. Such speed-ups seem plausible as GPU’s increase in power (Huang’s Law).

## Methods

One of the successful algorithms in use at the present time for soft tissue is based on the paraxial approximation. This approximation assumes that the vast majority of acoustic signal is propagating forward, with relatively small angle scattering taking place at heterogeneities within the breast. However, the empirical evidence^13^ shows that the side scattering is sufficiently accounted for to yield high resolution reconstructions that are quantitatively accurate. This is thought to be due to the full tomographic nature and high redundancy of our collected data, and the particular implementation of the paraxial approximation, as well as to the particular frequencies chosen in the imaging protocol. A typical protocol starts with an initial estimate from a time of flight based inversion and creates a low resolution 3D image at 0.35 MHz (for example) using the full wave inversion method discussed below. The resulting image is used as the starting estimate for an image at (for example) 0.4 MHz. We proceed in a step wise fashion creating volumes at successive frequencies, e.g. 0.4, 0.5, …, 1.2, 1.3 MHz, i.e. increasing the frequency by 0.1 MHz successively. A ‘protocol’ involves choosing the intermediate frequencies and the number of iterations at each frequency. The total reconstruction time includes all iterations at all frequencies in the protocol and thus must be chosen carefully. The particular choice of frequencies and iterations is a subject of research presently and is designed to miminize the number and severity of local minima artifacts that can be introduced into the final image at 1.3 MHz.

It is important to note that the data acquisition (DA) is carried out in the time domain using a single chirp approximately 50 μsec long. After the time domain data has been acquired at all levels, views and receivers, it is subsequently Fourier transformed to the frequency domain and stored as complete data sets at each frequency. These complete data sets at each frequency are the raw data used in the reconstruction algorithm, as described above. Thus our transmit-receive system has a large fractional bandwidth of ~ 1.2. This means that the data at the lower frequencies is somewhat noisy, and therefore is used sparingly and in conjunction with high redundancy of our data. Some of these points are discussed more completely in Wiskin^13^.

Note that the present algorithm also produces images at about 1.3 MHz, utilizing frequencies ranging from 0.35 to 1.3 MHz. This is considered a very low range for medical ultrasound or whole body imaging. However, because the inversion algorithm is a model based optimization procedure, the contrast and spatial resolution are ~ $\lambda {/}\sqrt 2$^51^. In some sense this can be considered optimal and is obtained because all of the effects associated with wave propagation are accounted for: in particular, refraction, diffraction and multiple scattering. The paraxial approximation limits the model somewhat. However, it is found a posteriori that the particular data acquisition employed here is compatible with transmission model we employ. Therefore the characteristics of the received wave and the modeled wave resulting from modelling propagation through the breast agree.

**Figure 11:**
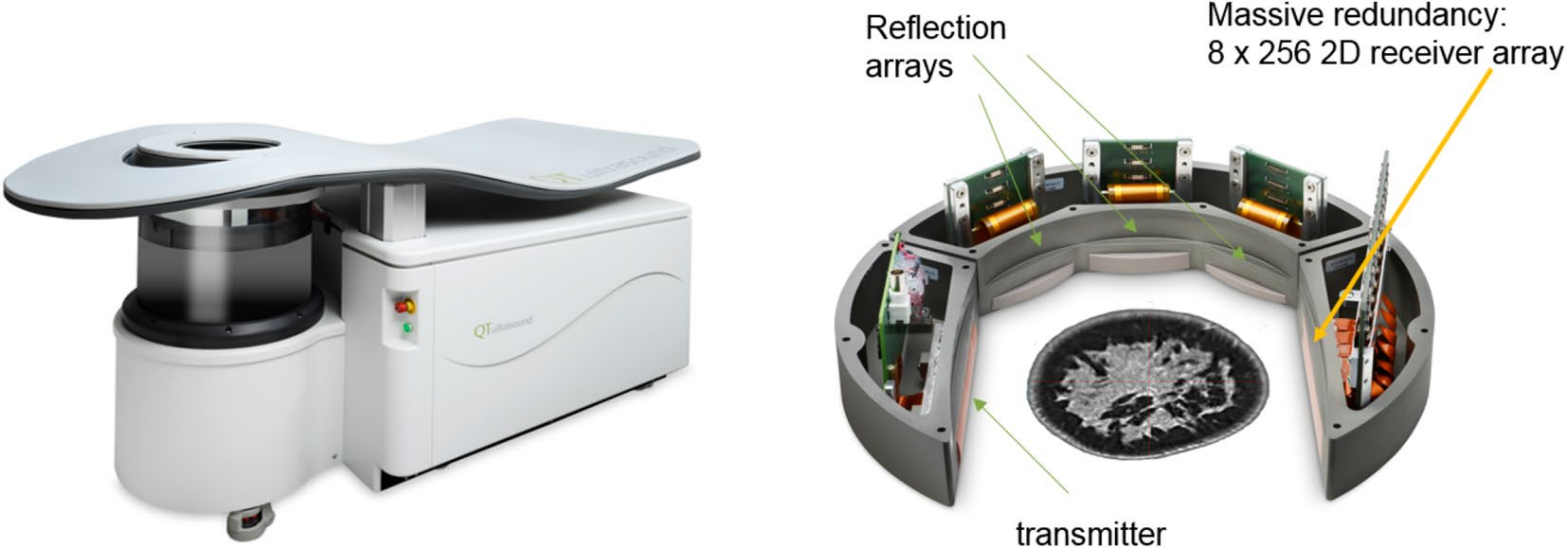
Breast Imaging Device from QT Ultrasound, LLC. Close view of the transmission receiver array pair, and the three (3) reflection mode transceivers used in the scanner. The array housing rotates 360° in the water bath, then moves up 2 mm and rotates in the opposite direction. One of the reflection arrays shoots every 2°, and the transmission receiver array system shoots and receives every 2°.

**Figure 12:**
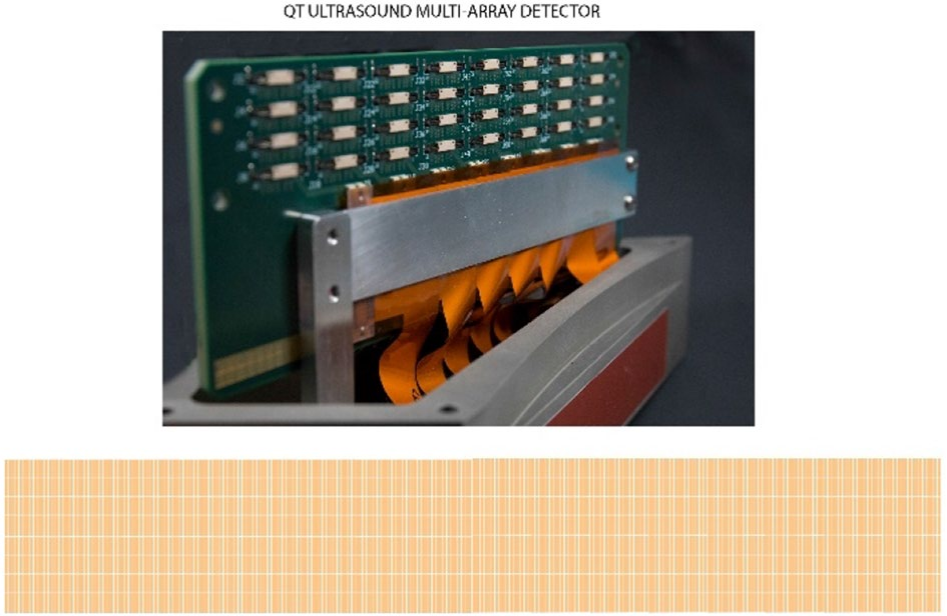
Close up view of the receiver array with 2048 individual array elements in 8 rows and 256 columns to collect fully 3D data to be used in the fully 3D reconstruction algorithm. The 3D nature and redundancy of the data collected by this design may be instrumental in the success of the inversion in this scenario.

The process of modeling wave propagation and predicting the received field when a known acoustic (or electromagnetic) wave impinges on a known object is “*forward scattering*”. The dual process of *measuring* the received field, and thereby predicting the characteristics of the unknown object is known as “*inverse scattering*”. This is accomplished in our case essentially through a large scale minimization with ~ 40 million unknowns, and an objective function which essentially represents the magnitude (squared sum) of the mismatch between the predicted field at the receiver elements and what is actually measured there, for all levels, views and receiver elements, by our device as shown in Figs. 11 and 12^13^.

This large-scale minimization requires the solution of a partial differential equation used to model wave propagation, and closely related ‘adjoint’ equations, multiple hundreds of times. This has been reported elsewhere^13,38,64^, and the exact determination of which frequencies to use and iteration counts is still a very active area of investigation. See also Wiskin et al.^13^.

Based on its success the method has been extended to clinically important situations where bone are present, despite the fact that it is the conventional wisdom in the ultrasound community that the presence of large contrast media, such as bone, will cause substantial artifacts in the reconstructed speed of sound (SOS) map. Furthermore, it has been believed in the past that the only way to address this artifact problem is by using the full Helmholtz wave equation that governs acoustic wave propagation in inhomogeneous media. The difficulty with this is the elliptic nature of the Helmholtz partial differential equation (PDE). Such PDE’s, when discretized for solution on the computer, involve the inversion of very large matrices, which is a notoriously time consuming, and clinically relevant images must be available in tens of minutes—not days^1^.

### Data acquisition

The ultrasound scanner consists of a plane wave transmitter, a 2048 element receiver array, and 3 adjunct reflection arrays in a water bath as in Figs. 11 and 12. The array rotates through 360 degrees shooting a pseudo-plane wave every two degrees and intermittently and simultaneously the 3 reflection arrays take turns emitting 192 beam-formed signals and receiving them (also beam-formed) every two degrees. The resulting signal is Fourier transformed and stored as frequency domain data as the reconstruction algorithm is carried out in the frequency domain.

### Data processing: quantitative inverse scattering (full wave inversion)

We have developed a fully 3D inverse scattering algorithm that incorporates the 3D nature of the acoustic field, and a concomitant refraction corrected (3D) reflection algorithm that incorporates the speed of sound (SOS) map generated by the inverse scattering reconstruction^13^.

For the transmission inversion we use a model based large scale minimization at a particular angular frequency $\omega_{j}$ based on an L_2_ minimization. The data residual is defined as the difference between the predicted and measured data at a particular angular frequency $\omega_{j}$:

1 $${\mathbf{r}}_{{\omega_{j} \theta }}^{l} \left( \gamma \right) \equiv \left( {{\hat{\mathbf{d}}}_{{\omega_{j} \theta }}^{l} \left( \gamma \right) - {\mathbf{d}}_{{\omega_{j} \theta }}^{l} } \right) \in {\mathbf{C}}^{{N_{R} }}$$

where the vector **r** encapsulates the difference at each vertical level *l, and* azimuthal angle *θ*, for a particular angular frequency ω = $\omega_{j}$. Here the object function $\gamma \left( {\mathbf{x}} \right) \equiv \left( {\frac{{c_{0} }}{{c\left( {\mathbf{x}} \right)}} - 1} \right) - i\frac{{\alpha_{dB} \left( {\mathbf{x}} \right)}}{{f_{j} }}\frac{{c_{0} }}{2\pi *8.686}$, where $c_{0}$ is the background (water) speed of sound, $c\left( {\mathbf{x}} \right)$ is the spatially varying speed of sound, $\alpha_{dB} \left( {\mathbf{x}} \right)$ is the spatially varying attenuation of sound in dB, and $f_{j}$ is the linear frequency given by: $\omega_{j} = 2\pi f_{j}$. More details are in Wiskin et al.^13^. The key in the reconstruction is the 3D nature of the data collected. See Fig. 12: all levels of data affect a given level of the image, and conversely all levels of the image are affected by one level of data. This is due to the 3D nature of the algorithm.

### Extension of imaging in the presence of bone

Bone has historically presented substantial problems to ultrasound images, causing severe artifacts and degradation of image quality due to the high impedance contrast that they engender. Utilizing special ‘sequences’, i.e. appropriate frequencies and iteration counts, we have achieved quantitatively accurate speed of sound images even in the presence of bone. The lack of artifacts around these high impedance volumes is unique to this 3D ultrasound tomographic technique and may be due to the high redundancy and 3D nature of the data.

### Reflection image formation

While the inverse scattering solution for quantitative transmission tomography is more sophisticated mathematically, the refraction corrected reflection image supplies important high resolution images of the connective tissue. This high-resolution image shown in Fig. 1, reflection fused with the quantitative transmission SOS image, is critical in showing detail that is missed in the SOS image. The basis for the refraction correction is the solution of the eikonal equation which models wave propagation at infinite frequencies:

2 $$\left| {\nabla \phi \left( {\mathbf{x}} \right)} \right| = n$$

Here $n\left( {\mathbf{x}} \right) \equiv {{c_{o} } \mathord{\left/ {\vphantom {{c_{o} } {c\left( {\mathbf{x}} \right)}}} \right. \kern-\nulldelimiterspace} {c\left( {\mathbf{x}} \right)}}$ *is* the index of refraction, c is the speed of sound, and *ϕ* is the phase function for the wave, i.e. the total field $u\left( {\mathbf{x}} \right) \equiv A\left( {\mathbf{x}} \right)e^{{ - ik_{o} \phi \left( {\mathbf{x}} \right)}}$, $k_{o} \equiv {\omega \mathord{\left/ {\vphantom {\omega {c_{o} }}} \right. \kern-\nulldelimiterspace} {c_{o} }}$, where $k_{o}$ is the free space wave number, $c_{o}$ is the free space speed of sound, *A* is an amplitude function and $\omega$ is the angular frequency. The method of lines is used to convert Eq. (2) to a system of ODE’s

$$\frac{d}{ds}\left( {n\frac{{d{\mathbf{r}}}}{ds}} \right) = \nabla n$$

With wavenumber $k\left( {\mathbf{x}} \right) \equiv \omega /c\left( {\mathbf{x}} \right) - i\alpha \left( {\mathbf{x}} \right)$, where $c\left( {\mathbf{x}} \right)$, $c_{o}$ are the spatially dependent and background speeds of sound, and α is the attenuation. This algorithm is also based on modeling an approximation to wave propagation through tissue. However, in this case the modeled and transmitted/reflected energy is at a higher frequency (~ 4 MHz). Note that this is still considered low frequency for medical imaging. See Refs^64,65^ for a description of this procedure.

## Supplementary information

Supplementary Information 1.

Supplementary Information 2.

Supplementary Information 3.
